# Tornado cleaning technique for food impaction in gastroduodenal stent using a high-rotational spiral basket catheter

**DOI:** 10.1055/a-2658-0539

**Published:** 2025-08-19

**Authors:** Takehiko Koga, Keisuke Matsumoto, Yi-Ling Ko, Makoto Fukuyama, Naoaki Tsuchiya, Yusuke Ishida, Fumihito Hirai

**Affiliations:** 1 38068 Department of Gastroenterology and Medicine, Fukuoka University Faculty of Medicine, Fukuoka, Japan

Food impaction can cause endoscopic gastroduodenal stent (GDS) occlusion, requiring endoscopic cleaning 1 2 . However, removing the residue stuck to the GDS lumen is often difficult. Recently, a novel basket catheter became available in Japan that is reportedly useful for removing bile duct stones 3 4 5 . This catheter comprises an eight-wire spiral basket connected to a rotating handle using a four-layer catheter shaft with a high torque response ( Fig. 1 ). Herein, we report on an efficient GDS cleaning technique using this catheter ( Fig. 2 , Video 1 ).

**Fig. 1:**
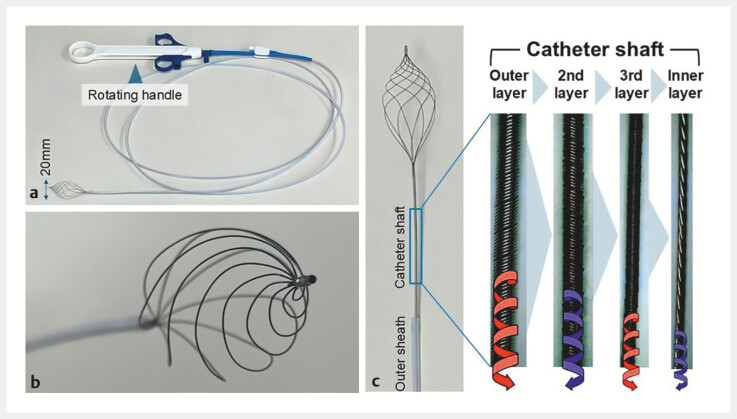
Structure of the novel basket catheter. **a** The basket catheter has a rotation function. The width of the basket is 20 mm. **b** The basket is formed by eight spiral wires. **c** The rotating handle and basket are connected using a special catheter shaft. The catheter shaft is composed of four layers of coil springs, and the direction of the springs alternates for each layer. This structure allows for a high torque response.

**Fig. 2:**
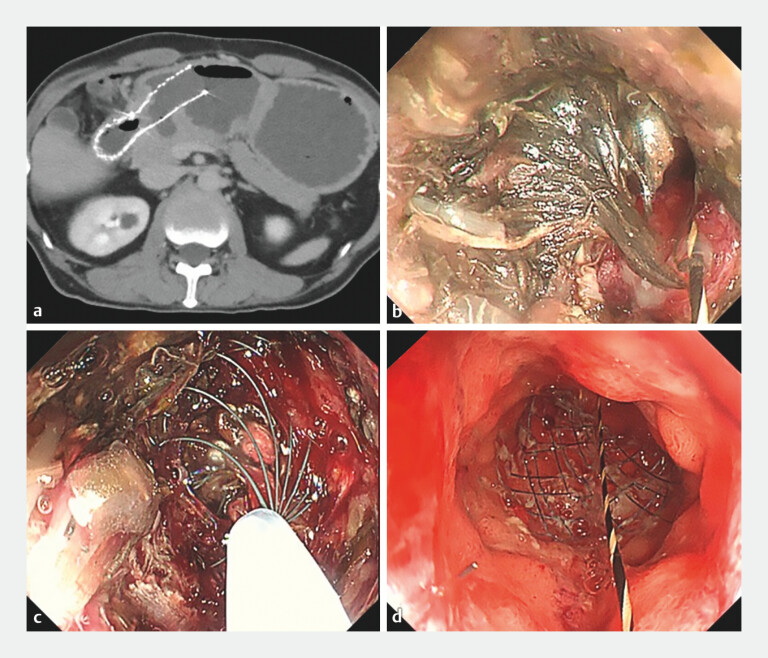
Gastroduodenal stent cleaning using the novel basket catheter. **a** Computed tomography three weeks after gastroduodenal stent placement: the stomach is distended and the stent and stomach are filled with residue. **b** Endoscopic findings: the stent is occluded by food impaction. **c** Stent cleaning with the novel basket catheter. **d** Endoscopic findings after cleaning: the stent was successfully re-opened.

Tornado cleaning technique for food impaction in gastroduodenal stent using a novel basket catheter.Video 1

A 63-year-old man who underwent a cholecystectomy for gallbladder cancer developed a gastric outlet obstruction (GOO) due to local recurrence involving the duodenal bulb. The GOO improved on performing endoscopic GDS placement (HANAROSTENT Naturfit; Boston Scientific, Marlborough, Massachusetts, USA) in the duodenal stenosis. However, frequent vomiting developed three weeks postoperatively. Computed tomography revealed residual fluid in the GDS lumen and a distended stomach; therefore, endoscopic re-intervention was performed. The stomach contained substantial residue, and the GDS was occluded because of food impaction. Water jet cleaning barely removed any food residue on the stent mesh. Consequently, we cleaned the GDS using a basket catheter (RASEN2; KANEKA Medical, Osaka, Japan) in a procedure we termed the “tornado cleaning technique” due to its rapid, spiral motion resembling a vortex.

First, a guidewire was advanced through the GDS lumen, and the basket catheter was inserted into the GDS under guidewire guidance. Then, the basket catheter was rotated into the residue at a high speed, and the basket wires efficiently removed the residue. Most food residue was successfully removed, and the GDS lumen was re-opened. Following this procedure, the patient was able to eat without any adverse events. No additional intervention was required, and the patient was discharged.

In conclusion, the tornado technique using the basket catheter is useful for rapidly and safely cleaning the GDS lumen.

Endoscopy_UCTN_Code_TTT_1AO_2AL

## Notes

Endoscopy E-Videos https://eref.thieme.de/e-videos
*E-Videos* is an open access online section of the journal *Endoscopy* , reporting on interesting cases and new techniques in gastroenterological endoscopy. All papers include a high-quality video and are published with a Creative Commons CC-BY license. Endoscopy E-Videos qualify for HINARI discounts and waivers and eligibility is automatically checked during the submission process. We grant 100% waivers to articles whose corresponding authors are based in Group A countries and 50% waivers to those who are based in Group B countries as classified by Research4Life (see: https://www.research4life.org/access/eligibility/ ). This section has its own submission website at https://mc.manuscriptcentral.com/e-videos .
